# A Dynamic Task Allocation Framework in Mobile Crowd Sensing with D3QN

**DOI:** 10.3390/s23136088

**Published:** 2023-07-01

**Authors:** Yanming Fu, Yuming Shen, Liang Tang

**Affiliations:** School of Computer and Electronic Information, Guangxi University, No. 100, University East Road, Nanning 530004, China; ymfu@gxu.edu.cn (Y.F.); 2113591016@st.gxu.edu.cn (L.T.)

**Keywords:** mobile crowd sensing (MCS), deep reinforcement learning (DRL), dueling dqn, double deep Q network (D3QN), multi-objective, dynamic task allocation

## Abstract

With the coverage of sensor-rich smart devices (smartphones, iPads, etc.), combined with the need to collect large amounts of data, mobile crowd sensing (MCS) has gradually attracted the attention of academics in recent years. MCS is a new and promising model for mass perception and computational data collection. The main function is to recruit a large group of participants with mobile devices to perform sensing tasks in a given area. Task assignment is an important research topic in MCS systems, which aims to efficiently assign sensing tasks to recruited workers. Previous studies have focused on greedy or heuristic approaches, whereas the MCS task allocation problem is usually an NP-hard optimisation problem due to various resource and quality constraints, and traditional greedy or heuristic approaches usually suffer from performance loss to some extent. In addition, the platform-centric task allocation model usually considers the interests of the platform and ignores the feelings of other participants, to the detriment of the platform’s development. Therefore, in this paper, deep reinforcement learning methods are used to find more efficient task assignment solutions, and a weighted approach is adopted to optimise multiple objectives. Specifically, we use a double deep Q network (D3QN) based on the dueling architecture to solve the task allocation problem. Since the maximum travel distance of the workers, the reward value, and the random arrival and time sensitivity of the sensing tasks are considered, this is a dynamic task allocation problem under multiple constraints. For dynamic problems, traditional heuristics (eg, pso, genetics) are often difficult to solve from a modeling and practical perspective. Reinforcement learning can obtain sub-optimal or optimal solutions in a limited time by means of sequential decision-making. Finally, we compare the proposed D3QN-based solution with the standard baseline solution, and experiments show that it outperforms the baseline solution in terms of platform profit, task completion rate, etc., the utility and attractiveness of the platform are enhanced.

## 1. Introduction

In recent years, the rapid development of technologies such as the Internet of Things, micro-sensors and 5G, and the increased demand for smart city sensing has contributed to the boom of a new sensing paradigm known as mobile crowd sensing (MCS) [1,2]. Mobile crowd sensing is a new large-scale sensing paradigm that mainly operates through the participation of a large number of users to obtain sensing data. People can use their mobile phones or smart devices to perform complex and large-scale sensing tasks [3], thus forming a large-scale, anytime, anywhere sensing system which is closely related to people’s daily lives. A typical crowd-sensing system usually consists of workers, users and the platform. The user posts tasks on the platform and the platform distributes the collected sensing tasks to the workers, who complete the sensing tasks and get paid. MCS research mainly includes data quality management [4,5], privacy protection [6,7], incentive design [8,9], worker selection, task assignment [10,11], etc. MCS also has a wide range of application scenarios, such as traffic planning [12], indoor positioning [13,14], energy management [15], air monitoring [16], public safety [17], etc. Mobile crowd sensing has now become an effective method for meeting the needs of large-scale sensing applications [18], such as sensor networks for detecting bridge vibrations on mobile vehicles [19], Creekwatch for monitoring the condition of river basins [20] and crowd engagement systems for public transport arrival time prediction [21].

One of the central issues in mobile crowd sensing is task allocation. Assigning tasks to specific workers under certain conditions not only increases the effectiveness of the platform, but also reduces worker consumption. Hence, ensuring appropriate task allocation is a mutually beneficial strategy for both participants and the platform. At this stage, task assignment studies can be divided into two categories based on the degree of worker participation: participatory sensing task assignment [22] and opportunistic sensing task assignment [23]. In the context of opportunistic sensing task assignment, workers perform sensing tasks along a predetermined route. However, in the case of participatory perception, workers are required to generate their own movement routes based on the tasks assigned by the platform. The strategies for task assignment heavily rely on the temporal and spatial information associated with both the workers and the tasks. This poses a challenge in dynamically assigning tasks to workers while considering spatio-temporal aspects.

In addition, for task assignment issue, it can be divided into user-centric [24] and platform-centric modes [25] according to different task assignment methods. In the user-centric model, workers are more autonomous and can choose which tasks to complete themselves by browsing tasks posted on the server and then uploading the collected data through their smart devices. As it relies mainly on workers’ autonomy to select tasks, the worker-centric model has some drawbacks, such as that individual workers tend to consider only their own interests and select only high-value tasks, and there may even be a situation where pending selection of tasks can go to completion but multiple workers compete for a high-value task, resulting in an unbalanced distribution of sensing tasks, making it difficult to maximise worker resources to complete more tasks and improve the quality of task execution. In contrast, in the platform-centric model, the platform collects all information from the demand of sensing tasks (such as response time and completion quality) and workers, and can use global optimisation algorithms to allocate tasks to different workers according to different strategies, making good use of worker resources while completing the task assignment in a global manner.

In MCS applications, excluding tasks with complex requirements, sensing tasks can be divided into two types: area tasks and point tasks, depending on the size of the sensing range [26]. Therefore, workers must reach these locations in order to perform their assigned tasks. For each worker, the reward received is related to the path taken to perform the task, and the order in which the tasks are performed directly affects the worker’s travel path, so the task assignment problem can be considered as a path planning problem. However, even if there is only one worker, the path planning problem without various constraints is computationally intractable, similar to the travelling salesman problem [27]. To plan the worker’s travel path, the platform makes decisions on the worker’s sensing task assignment process sequentially. Since reinforcement learning [28] has a long-term perspective, considers long-term payoffs and is particularly suited to making sequential decisions, it is promising for RL to be applied to task assignment problems.

Thus, since the platform is seen as an agent for RL, the status of sensing tasks and workers is regarded as the environment of RL. However, when the state and action space is large, traditional RL methods (e.g., Q-learning [29]) suffer from slow convergence and dimensional disasters. To address this problem, deep reinforcement learning (DRL) has introduced deep learning methods to RL and has already made great progress in several areas, such as natural language processing [30] and recommender systems [31]. In this paper, an MCS framework is proposed with five phases. Initially, data requesters (platform users) post their sensing tasks and relevant information (e.g., location, time window and budget) in the platform. Secondly, the platform announces the reward rules to the workers and filters the set of optional workers. Thirdly, the platform solves the task allocation problem and plans the travel path for the recruited workers. Then, the recruited workers move along the planned path to perform their assigned tasks and then upload their information (e.g., location, estimated completion time, etc.) to the platform, which feeds the information back to the user. Finally, the platform validates the data collected and sends it to the corresponding user. At the same time, the platform distributes rewards to the recruited workers. In existing studies, usually the platform-centric task allocation model only considers the interests of the platform [32], ignoring the feelings of other participants, which is not conducive to the development of the platform. Therefore, a weighted multi-objective approach was adopted, taking into account both the participants’ experience and the platform’s profit. To achieve this goal, we proposed a D3QN solution with a double deep Q network (DDQN) structure and a Dueling architecture for the task allocation problem. In addition, three baseline solutions are considered for performance comparison, namely DQN, ε–greedy and random solutions. Finally, we evaluate our proposal experimentally.

In brief, the main contributions of this paper are summarised as follows:

- We propose a time-sensitive dynamic multi-objective task assignment problem and use a new MCS framework to efficiently recruit workers and have them complete their assigned sensing tasks within a specified time;
- We propose an MCS task assignment strategy based on a modified version of DQN. Considering the location of workers, time constraints of tasks and other factors, it is closer to realistic scenarios. To our knowledge, we are the first to use D3QN to solve the task allocation problem for MCS;
- We use three baseline solutions (i.e., DQN, ε–greedy and random solutions) and conducted various simulations with different numbers of workers to evaluate the performance of the algorithm. The results show that our proposed D3QN solution outperforms the baseline solutions;
- We use weighted multi-objectives to optimise multiple objectives, comprehensively consider platform’s profit and participants’ experience and achieve good results, improving the practicability of the platform.

The remainder of this paper is organised as follows. Section 2 presents related work, Section 3 describes the structure of the task allocation framework and the formulation of the task allocation problem, and Section 4 details the D3QN solution and the three baseline solutions. Section 5 presents the simulation scenarios and results. Finally, Section 6 concludes the paper.

## 2. Related Work

### 2.1. Task Allocation of MCS

A key issue in MCS is task allocation, where the goal is to assign sensing tasks to eligible workers. The task allocation problem in MCS has been studied from many perspectives throughout the literature [33,34,35,36]. Different criteria [37,38] can be used to classify them into different categories, e.g., existing research work can be divided into two categories based on the level of worker participation in MCS systems: opportunistic and participatory models. The sensing quality of tasks depends heavily on the daily trajectories of workers [39] and is unstable, as there is less interference with workers in the opportunistic model and platform organisers assign tasks mainly through their historical action trajectories. In addition, task assignment strategies based on chance patterns usually require prediction of workers’ trajectories, and, although different trajectory prediction algorithms [40,41] have been proposed and proven to be effective to some extent, the accuracy cannot be theoretically guaranteed due to complex and unpredictable realistic conditions, which have a significant impact on the final sensing quality of task assignment. Ding et al. [42] proposed a dynamic delayed decision user recruitment problem, and designed three algorithms, among which, semi-Markov prediction algorithm and prediction algorithm for meta-paths were used for user selection to maximise user utility, and delayed decision-based task assignment algorithm determined the time point of task assignment and the set of assigned tasks with high task completion rate, budget utilisation and user diversity. Guo et al. [43] investigated the multi-task MCS environment by considering two scenarios of whether workers change their movement trajectory to actively engage in perception and proposed the ActiveCrowd task assignment framework, which, for time-sensitive tasks, requires workers to intentionally move to the task location to minimise the total movement distance. For delay-tolerant tasks, workers whose paths are expected to pass through the task site are selected to minimise the total number of workers. Finally, two greedy augmented genetic algorithms are introduced to address this problem across different scenarios.

The participationist model requires workers to change their original life trajectory and move to a specific place to complete a task. Liu et al. [44] developed a participant selection framework, TaskMe, which considers two different multi-task allocation scenarios. For FPMT (few participants, many tasks), two algorithms based on minimum cost maximum flow theory were proposed to maximise the total number of tasks completed while minimising the total distance travelled. For MPFT (many participants, few tasks), two algorithms based on multi-objective optimisation theory were proposed to minimise the total incentive payoff and the total distance travelled. Estrada et al. [45] proposed a service computing framework for time-constrained task allocation in a location-based crowd-aware system to maximise the quality of information aggregation, reduce the budget and response time for performing tasks and improve the reputation of average recommenders and their payoffs.

In addition, in existing studies, platform benefits, data quality and sensing costs are only considered from a single perspective of the platform or the participant, and there is always one side of the concern that is overlooked. Therefore, in this paper, we take into account the benefits of the platform and the experience of the participants and consider the task allocation of MCS from a holistic perspective, with the goal of maximising platform profit while minimising workers’ travel distance and response time received by users. Furthermore, in some of the studies, the travel time and distance of workers were not clearly defined. For example, Gong et al. [46] considered the movement path of workers to maximise the sum of task quality but did not consider the actual travel time versus distance. This is a serious problem when considering time-sensitive tasks in MCS applications. Therefore, to increase the usefulness of the model and to better fit the real-world scenario, we defined the travel speed of each worker and the time limit of the sensing task, considering constraints on the total budget, the maximum distance the worker can move and the task response time.

### 2.2. Deep Reinforcement Learning

Reinforcement learning is a subset of machine learning [47], unlike supervised learning [48], which requires labelled training data to train a model. Reinforcement learning mainly explores the environment through the agent autonomously, takes actions to act on the environment and obtains feedback from the environment. It also learns how to make decisions in ideal situations so as to maximise the cumulative reward. Traditional reinforcement learning primarily relies on a tabular approach, wherein action-state values of discrete environments are stored in table format. However, this approach has limitations, as it becomes cumbersome to handle large numbers of states and actions. Q-tables can grow significantly in size, requiring extensive time and storage resources for search and storage and are susceptible to the curse of dimensionality.

Therefore, to solve this problem, we used DQN and its improved algorithm. DQN proposed by Google DeepMind [49,50] is a kind of well-known deep reinforcement learning. In the DRL method, DQN is widely used to approximate Q-values, replacing the Q-value table in traditional RL methods. Among them, deep learning [51] is used to enhance RL, and DRL has been applied in many fields [52,53]. Mittal et al. [54] used a graph convolutional neural network to model the graph structure and solved a large-scale minimum vertex coverage problem and a maximum coverage problem using DQN and a greedy strategy, obtaining an improvement in optimisation power compared to previous models. Dai et al. [55] proposed an AI-enabled vehicular network architecture that can intelligently orchestrate edge computation and caching. In addition, a joint edge computing and caching problem was proposed and the deep reinforcement learning method DDPG was used to maximise the system utility. Dong et al. [56] proposed a new DQN-based task scheduling mechanism in a cloud manufacturing environment to reduce task execution time by scheduling tasks with priority relationships to cloud servers. Liu et al. [57] proposed a new DRL-based UAV control method based on the recent actor–critic approach to maximise the energy efficiency function taking into account communication coverage, fairness, energy consumption and connectivity, and extensive simulation results also demonstrated its effectiveness, robustness and superiority in terms of various metrics.

Our MCS task allocation problem is a combinatorial optimisation problem, satisfying various optimisation objectives and constraints. This is usually NP-hard, and traditional greedy or heuristic algorithms suffer from poor robustness and high computational cost. Therefore, we wish to use reinforcement learning, a novel approach, to solve the task allocation problem for MCS. Finally, to further improve the performance of DQNs, various techniques are used, such as Double DQN [58] and Dueling DQN [59]. Compared with other methods, our D3QN method has shown better performance in terms of learning efficiency, convergence and experimental results.

## 3. System Model and Problem Formulation

### 3.1. System Model

**Definition** **1** (Sensing tasks, *V*)**.** *Sensing tasks are published by platform users and received by the platform to record information about the tasks and recruit workers for them. The set of sensing tasks is denoted by V=$\left\{v_{1},v_{2},\ldots ,v_{m}\right\}$, a single task is denoted as $v_{i}$ and the budget provided by the user for task $v_{i}$ is denoted as $b_{i}$. The location of task $v_{i}$ is $l_{i}$, a point in the 2D space. In addition, we assume that each sensing task $v_{i}\in V$ is time-sensitive and can only be completed within a time window $\left[t_{i},t_{e}^{i}\right]$. During the time window, the task can be completed once the recruited worker moves to the corresponding area.*

**Definition** **2** (Workers, W)**.** *The set of workers is defined as $W=\{w_{1},w_{2},\ldots ,w_{n}\}$, and the set of feasible workers filtered by the platform for task $v_{i}$ based on the current state is denoted as $W^{i}$, $W^{i}\in W$. In addition, a single worker is denoted as $w_{j}$, the time at which worker $w_{j}$ starts performing the task is $t_{j}$ and the movement speed is denoted as $f_{j}$. The arrival position of a worker is denoted as $l_{j}$ and the total movement trajectory is F, which can be tracked by a positioning technique (e.g., GPS).*

**Figure 1:**
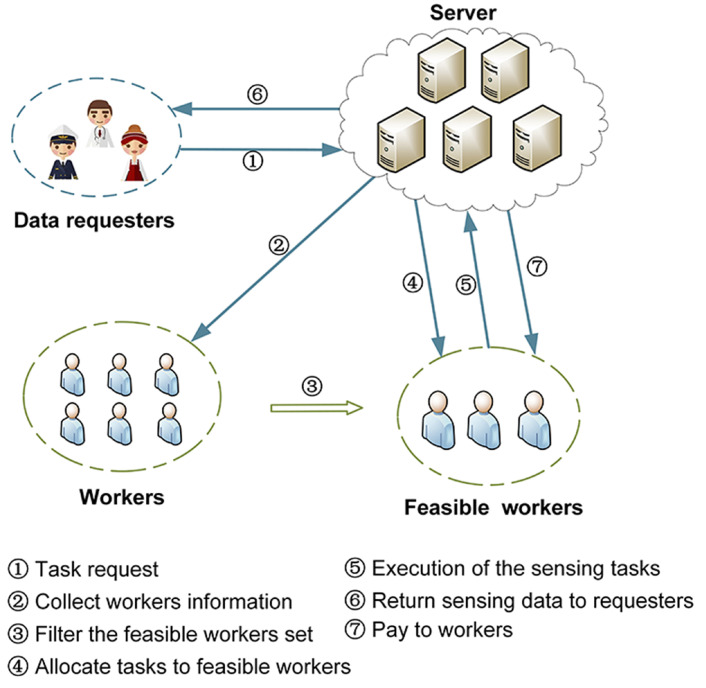
The proposed framework of task allocation in mobile crowdsensing.

**Definition** **3** (Time-sensitive dynamic task assignment problem, TS-DTA)**.** *Based on the above description of tasks and workers, combined with the task allocation framework of this paper, as shown in Figure 1. It is worth mentioning that our ideal application scenario is that workers can complete the MCS lightweight perception tasks initiated by users within a certain period of time under the condition of limited resources. The so-called lightweight, such as taking pictures or sending messages at designated locations, to monitor air pollution conditions. This type of task requires negligible completion time and resource consumption, and basically does not require workers’ resources and capabilities. The time-sensitive dynamic task assignment problem is stated as a five-step process as follows.*

The first step is the submission of the sensing tasks, where the users publish the sensing tasks to the platform on the user side and submit relevant information such as location, time, type, budget, etc. Then, the platform saves the task details and adds the task to the task queue. The second step is for the platform to filter the feasible workers set. After the task arrives, the detailed workers’ personal information such as location, unit distance quotes and average moving speed are uploaded to the platform. The platform primarily selects a feasible workers set based on factors such as the position of the workers, the speed of the workers, the current status of the workers and the distance between the workers and the tasks. The third step is task allocation by the platform. It means that the platform selects a reasonable allocation strategy through algorithms based on the current task and the users’ status, under several constraints such as the maximum distance the worker can move and the task response time. Eventually, the task details are sent to the worker and the worker completes the confirmation. The fourth step is the execution of the sensing tasks. In this stage, the worker moves in a straight line along the planned path to perform the assigned sensing task (This means to travel to the mission point in a straight line regardless of factors such as obstacles on the way). During the execution of the task, the worker uploads the current geographical location, task progress and other relevant information to the platform. The platform then feeds this information back to the user for easy viewing in order to enhance the user experience. After completing the task, the worker uploads the collected data to the platform, which can apply certain techniques (e.g., machine learning methods) to validate and evaluate the uploaded data. The last is the sending of perception data and workers’ reward. The platform sends the collected data to the users and pays rewards to workers who have completed the tasks. This is the final stage of the current sensing cycle, and the platform then starts the next round of sensing activity, and so on, until an optimal strategy is found. Ultimately, the goal of the TS-DTA problem is to improve platform profit and participant experience by combining multiple objectives under constraints of optimisation within cost and time budget constraints. Table 1 shows notations that are used in the rest of the article.

### 3.2. Problem Formulation

The goal of the multi-objective dynamic task assignment problem is to give a set of available workers when tasks arrive randomly. Through a reasonable allocation strategy, the multi-objective, comprehensive optimisation is realised: maximise the platform’s profit while minimising the worker’s travelling distance and the response time of the workers obtained by the users.

First, the platform in MCS acts as an intermediary between users and recruited workers. It can profit from its brokerage services by assigning sensing tasks to the recruited workers. Thus, the platform’s profit is denoted as P, the difference between the task budget deposited by the data requester and the salary paid to the recruited workers. On the one hand, users provide the platform with an advance payment and expect that the tasks they post will be completed within the budget. On the other hand, the recruited workers are paid by the platform when they complete these tasks. In general, the workers’ pay depends mainly on the distance of their travel path and the energy cost of completing the assigned tasks. Here, we assume that the tasks completed are light-weight tasks, such as taking pictures or sending messages at the assigned locations and monitoring the pollution status of the air. The energy cost of the workers is negligible compared to their travel cost. In this case, the travel cost of workers is more important than the energy cost. Therefore, the wage paid to each worker is defined as follows:

(1) $$p_{j}=\sum_{v_{i}\in F_{j}}\left(\theta_{j}\cdot d\left(F_{j}^{i}\right)\right)$$

where the coefficient $\theta_{j}$ denotes the unit payoff of the distance travelled to worker $w_{j}$ and $F_{j}^{i}$ is the travel trajectory between worker $w_{j}$ and task $v_{i}$. $d\left(F_{j}^{i}\right)$ is the distance between worker $w_{j}$ and the completed task $v_{i}$, wherein we use the Euclidean distance, derived from the latitude and longitude coordinates of the workers and tasks according to the Haversine formula:

(2) $$d\left(F_{j}^{i}\right)=2r\arcsin\left(\sqrt{\sin^{2}\left(\frac{{\mathrm{lat}}_{v_{i}}-{\mathrm{lat}}_{w_{j}}}{2}\right)+\cos\left({\mathrm{lat}}_{w_{j}}\right)\cos\left({\mathrm{lat}}_{v_{i}}\right)sin^{2}\left(\frac{{\mathrm{lng}}_{v_{i}}-{\mathrm{lng}}_{w_{j}}}{2}\right)}\right)$$

where *r* is the radius of the Earth, while ${\mathrm{lat}}_{v_{i}}$, ${\mathrm{lng}}_{v_{i}}$, ${\mathrm{lat}}_{w_{j}}$, ${\mathrm{lng}}_{w_{j}}$ are the latitude and longitude coordinates of the workers and the tasks, respectively. With this, we define the distance travelled by a worker during a complete task assignment as below:

(3) $$D=\sum_{v_{j}\in V,w_{j}\in W^{i}}\;d\left(F_{j}^{i}\right)$$

Next, we define the profit of the platform:

(4) $$P=\sum_{w_{j}\in W^{i}}\left(\sum_{v_{i}\in F_{j}}b_{i}-p_{j}\right)$$

This is the difference between the budget given by the users for the tasks and the wages paid to the workers. Here, $b_{i}$ is the payoff paid by the user for task $v_{i}$, $\sum_{v_{i}\in F_{j}}b_{i}$ is the total budget of the sensing tasks that the platform receives from the users and has the workers perform along path $F_{j}$ and $p_{j}$ is the corresponding payoff paid to the worker $w_{j}$. The profit of the platform is the sum of the difference earned by all workers and their assigned tasks. It is worth noting that, in different MCS applications, the payment rules can be more complex to take into account various factors. Changes in payment prices will not affect the applicability of our proposed solution.

Furthermore, in order to consider the quality of service for users, we will also take into account the workers’ response time that tasks receive. The workers’ response time for each task is determined by the platform’s response time and the execution time allocated to the workers by the platform and is constrained by the task’s response time. If either the platform’s response time or the task’s execution time exceeds the task’s response time, the task assignment will be considered a failure. Then, the total workers’ response time is defined as follows:

(5) $$T=\sum_{v_{i}\in V,w_{j}\in W^{i}}\left(t_{j}^{i}-t_{i}\right)$$

where $t_{j}^{i}$ is the time when worker $w_{j}$ starts executing task $v_{i}$ and $t_{i}$ is the time when task $v_{i}$ arrives. Here the propagation time of the message through the network is ignored and the time when the task is submitted by the user is regarded as the time of arrival of the task. Ultimately, the multi-objective function is as follows:

(6) R=α∗(K1−D)+β∗(K2−T)+γ∗P

where *K*1 and *K*2 correspond to the constants of the variables D and T, respectively, and the purpose is to transform the minimisation problem into a maximisation. α, β and γ are weighting coefficients that sum to 1. In reinforcement learning, each decision made is rewarded with feedback from the environment, and the reward sum of a complete round is recorded as greward. Meanwhile, the greward we can assign to a complete task can be written down as *R*.

Finally, consider the interests of the platform along with the experience of workers and users. We define the dynamic multi-objective task allocation problem of maximising platform profit versus minimising workers’ travel distance and workers’ response time:

(7a) Max.R

(7b) $$\;s.t.\;t_{j}\leq t_{r}^{i},\forall w_{j}\in W,v_{i}\in V$$

(7c) $$t_{j}+d\left(F_{j}^{i}\right)/f_{j}\leq t_{e}^{i},\forall w_{j}\in W,v_{i}\in V$$

(7d) $$\sum_{v_{i}\in V}pi\leq B$$

(7e) $$d\left(F_{j}^{i}\right)\leq d_{\max},\forall w_{j}\in W,v_{i}\in V$$

(7f) $$\sum_{w_{j}\in W}1\left(v_{i}\in F_{j}^{i}\right)\leq 1,v_{i}\in V$$

Here, the objective function is defined in (7a). Constraints (7b) and (7c) are time constraints that guarantee that each recruited worker $w_{j}\in W$ completes the planned path before the end of the task time, where $t_{j}$ is the time when the worker starts the task, $t_{r}^{i}$ is the workers’ response time of the task, $t_{e}^{i}$ is the end time of the task and $f_{j}$ is the movement speed of the worker. The constraint (7d) implies that the profit of the platform is positive. Next, for any worker $w_{j}\in W$ and task $v_{i}\in V$, the constraint in (7e) dictates that the length of the path cannot be greater than the workers’ maximum travel distance $d_{max}$. Finally, constraint (7f) implies that each task is assigned to at most one worker. Above, 1(·) is an indicator function that equals 1 when the conditions in the function parameters are satisfied.

## 4. Solutions of Formulated Problem

### 4.1. Markov Decision Process and Reinforcement Learning

In this section, first, the task assignment problem of MCS is considered from a reinforcement learning perspective, and then a D3QN-based solution is proposed. To evaluate the proposed solution, we also provide three baseline solutions as benchmarks, including DQN, ε–greedy and random solutions.

Reinforcement learning studies the sequential decision process by which an agent as the subject interacts with the environment as the object. Mathematically, it is generally normalised as a Markov decision Process (MDP), described by the current state and the action taken on it. A Markov decision Process can be described, where the next state is determined as $M=\langle S,A,R\left(s,a\right),P\left(s^{\prime },r|s,a\right),\eta \rangle$. In this case, *S* and *A* denote the finite state space and action space, respectively, and R(s,a) is the reward function. $P\left(s^{\prime },r|s,a\right)$ is the state transfer probability of obtaining the corresponding reward *r* from a given state and action to the next state. η∈[0,1] is the discount rate that responds to the importance of the current reward to future rewards. The goal of reinforcement learning is to maximise the cumulative reward $U_{t}=\Sigma_{\tau =t}^{T}\eta^{\tau }R_{\tau }$, where *T* is the number of steps for state *s* to reach the terminal state. $Q^{\pi }\left(s_{t},a_{t}\right)$ is then computed from the action-value function, which is defined as follows:

(8) $$Q^{\pi }\left(s_{t},a_{t}\right)=E\left[U_{t}|S_{0}=s_{t},A_{0}=a_{t}\right]$$

Through the function $Q^{\pi }$, it can be judged whether the policy function π performs the action $a_{t}$ under the state $s_{t}$ at time t. When maximising π with respect to $Q^{\pi }$, the optimal action value function can be obtained:

(9) $$Q^{*}\left(s_{t},a_{t}\right)=\max_{\pi }Q^{\pi }\left(s_{t},a_{t}\right)$$

Next, we treat the task assignment problem as a Markov decision process, represented by a five-tuple $M=\langle S,A,P,R,\eta \rangle$, based on an interaction model between the MCS server and the environment, where the platform is considered as the agent and *S* is a finite set of states, each consisting of the set of tasks observed by the current agent. *A* is a finite set of actions, with each action representing an assignment between a sensing task and a worker. *P* is the probability that the current agent will move to state s’ after taking action a in state *s*. *R* is the reward function, from which the value of the reward that can be obtained by moving from the current state to the next state is returned. η is the discount rate. It is worth noting that, for faster learning, we added penalties to all failed decisions. Thus, the reward function *r* for a round is defined as: follows

(10) $$r=\left\{\begin{array}{cc} \alpha *(K1-d)+\beta *(K2-t)+\gamma *p, & \mathrm{if}\;\mathrm{all}\;\mathrm{constraints}\;\mathrm{are}\;\mathrm{feasible}\\ \;\;\;\;z, & \mathrm{else} \end{array}\right.$$

where *K*1 and *K*2 correspond to the constants of the variables *d* and *t*, respectively, with the aim of transforming the minimisation problem into a maximisation one. *d* is the distance travelled by the performing worker, *t* is the workers’ response time for the current task, and  *p* is the profit that the platform can obtain from the current task. α, β and γ are weighting factors that sum to 1. *z* is a negative constant that is the penalty constant added for failed task assignment rounds.

Reinforcement learning problems can be solved in various ways, such as dynamic programming, Monte Carlo methods and temporal difference methods. Among them, the time difference method is favored because of its model-free characteristics. Q-learning is a typical algorithm of temporal difference methods and a widely used reinforcement learning method, which mainly focuses on estimating the value function of each state–action pair. For any state $s_{t}\in S$ and action $a_{t}\in A$ acquired at time *t*, Q-learning predicts the value of the state–action pair $\left(s_{t},a_{t}\right)$ by iteratively updating:

(11) $$Q\left(s_{t},a_{t}\right)\leftarrow Q\left(s_{t},a_{t}\right)+\sigma \left(R_{t+1}+\eta \cdot \max_{a^{\prime }}Q\left(s_{t+1},a^{\prime }\right)-Q\left(s_{t},a_{t}\right)\right)$$

where σ is the learning rate, η is the discount factor and $R_{t+1}$ is the reward obtained for the transition of the state from $s_{t}$ to $s_{t+1}$ after action $a_{t}$. $\max_{a^{\prime }}Q\left(s_{t+1},a^{\prime }\right)$ is the largest Q-value function of all possible actions in the new state $s_{t+1}$.

### 4.2. DQN Solution

DQN (Deep Q Network) is an approach that combines neural networks and Q-learning. The core of Q-learning is the Q-table, which is built to guide actions. However, this applies when the state and action space is discrete and not high dimensional. When the state and action space is high-dimensional, the Q-table will become very large and the amount of computer memory required to store the Q-table and the time consumed to find the state are both unacceptable.

Therefore, neural networks in machine learning were introduced to solve this problem. The neural network receives input from the state $s_{t}$ and, after analysis, outputs a Q-value vector of actions $Q(s_{t},\cdot ;\theta )$. θ is a parameter of the neural network and represents the weight between neurons. The DQN also uses two means of improving learning efficiency, namely, the experience replay mechanism and Fixed Q-targets. Experience replay refers to the use of buffer to store past and current experience, and, when the neural network parameters need to be updated, some random samples can be taken for learning. Thus, experience replay makes samples reusable and improves learning efficiency. The principle of Fixed Q-targets is to use two neural networks with the same structure but different parameters, where the parameters of the target network are $\theta^{-}$. Every certain number of steps, the parameters of the replicated neural network are used to update the target network. The Q values in the DQN are then updated by the following:

(12) $$Q\left(s_{t},a_{t};\theta \right)\leftarrow \left(1-\sigma \right)\cdot Q\left(s_{t},a_{t};\theta \right)+\sigma \cdot \left(r_{t}+\eta \cdot \max_{a}Q\left(s_{t+1},a\;;\theta^{-}\right)\right)$$

We then define the loss function in terms of the mean squared error as follows:

(13) $$\mathrm{Loss}\left(\theta_{i}\right)={\left(y_{i}-Q\left(s,a,\theta_{i}\right)\right)}^{2}$$

$$where\;y_{i}=\left\{\begin{array}{cc} r, & \;s^{\prime }\;\mathrm{is}\;\mathrm{the}\;\mathrm{termination}\;\mathrm{state}\\ r_{i}+\eta \cdot \max_{a}Q\left(s_{i+1},a\;;\theta^{-}\right), & s^{\prime }\;\mathrm{is}\;\mathrm{not}\;a\;\mathrm{termination}\;\mathrm{state}\; \end{array}\right.$$

where $y_{i}$ is the target action value function based on the action distribution of the output of the target Q-network. All parameters of the evaluated Q-network are denoted as $\theta_{i}$ at iteration generation *i* and are updated at each iteration. $\theta^{-}$ values come from the target Q-network; they are fixed and are only updated with $\theta_{i}$ every certain number of steps. η∈[0,1] is the discount factor, which determines the weights for considering long-term rewards.

### 4.3. D3QN Solution

Furthermore, the DQN algorithm itself is prone to overestimation. To address this issue, we adopted the double DQN approach, which involves training two Q-networks: the original Q-network and the target Q-network. The Q-network is responsible for action selection using parameter θ, while the target Q-network evaluates the action values using parameter $\theta^{-}$. Unlike the DQN method that directly selects the action with the highest value from the target Q-network for updating, the double DQN approach considers that the action with the highest value in the Q-network may not necessarily be the one with the highest value in the Dueling DQN target Q-network. This distinction allows for more effective mitigation of the overestimation problem while maintaining the algorithm’s performance.

**Figure 2:**
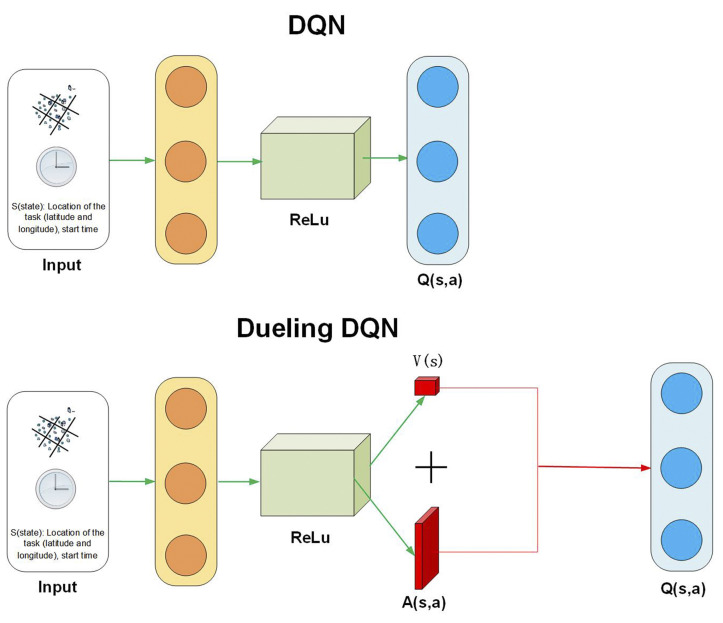
Network structure of DQN and Dueling DQN.

Then, Dueling is used to optimise the network architecture, as shown in Figure 2. The above is the traditional DQN, and the bottom is the Dueling DQN. In the original DQN, the neural network directly outputs the Q-value of each action. In contrast, the Dueling DQN decomposes the Q-value of each action into a V-value and a dominance function A(s,a), which can distinguish between what is rewarded by the state and what is rewarded by the action. The value function is then re-represented as follows:

(14) Q(s,a;θ,ω,φ)=V(s;θ,ω)+A(s,a;θ,φ)

where θ is the network parameter of the common part, ω is the network parameter of the unique part of the value function and φ is the network parameter of the unique part of the dominance function. In addition, in practice, for better stability, we replace the maximum operator with the average operator:

(15) $$Q\left(s,a;\theta ,\omega ,\varphi \right)=V\left(s;\theta ,\omega \right)+\left(A\left(s,a;\theta ,\varphi \right)-\frac{1}{\left|\;A\right|}\sum_{a^{\prime }}A\left(s,a^{\prime };\theta ,\varphi \right)\right)$$

Finally, D3QN updates the Q-value:

(16) $$Q\left(s_{t},a_{t};\theta \right)\leftarrow \left(1-\sigma \right)\cdot Q\left(s_{t},a_{t};\theta \right)+\sigma \cdot \left(r_{t}+\eta \cdot Q\left(s_{t+1},\underset{a}{\arg\max}Q\left(s_{t+1},a\;;\theta \right);\theta^{-}\right)\right)$$

Based on the above improvements, we propose a D3QN solution for the dynamic task allocation problem in crowd sensing. The algorithmic time complexity of the D3QN solution with experience replay, considering the training process, can be expressed as O(M*(N*(|U| + |V| + $W_{t}$))), where M is the maximum number of episodes, N is the number of samples in the experience replay, |U| denotes the size of the input state space, |V| denotes the number of actions and $W_{t}$ is the time spent processing a neural network layer for one worker and one task. The complexity of the algorithm mainly focuses on steps such as policy selection, action execution, experience playback, network update and task assignment. It should be noted that this complexity analysis is based on the training and update times of neural networks being far longer than other operations and may be affected by specific implementation details. Then, the pseudo-code for the D3QN-based dynamic task assignment algorithm is as described in Algorithm 1.

**Algorithm 1:** D3QN Solution with Experience Replay Solution
**Input:** 
V (tasks), W (users), N (capacity of replaymemory), ε (probability of random selection), M (maximum number of episodes), η (discount factor), C (frequency of updating target network)**Output:** 
R (greward of all episodes), {p,d,t} (profit of platform, travel distance of workers and workers’ response time), {$F_{j}:\forall w_{j}\in W$} (travelling trajectory of workers)
1:Initialise policy network Q with parameters θ2:Initialise target network $\hat{Q}$ with parameters $\theta^{-}$ = θ3:Initialise replay memory D with capacity of N4:R←−∞; // global maximum greward5:p,d,t←0; // profit, distance and response time6:**for** *k* = 1,M **do**7:    $R_{k}\leftarrow 0$; // local greward in current episode8:    $s_{t}\leftarrow s_{0}$; // initialise state9:    **while** $s_{t}\neq s_{e}$ **do**10:        Select $a_{t}=\arg\max_{a}Q^{*}\left(s_{t},a\;;\theta \right)$ with probability ε; otherwise, randomly select $a_{t}$11:        Execute $a_{t}$ to observe $r_{t}$ and $s_{t+1}$12:        Store $\left(s_{t},a_{t},r_{t},s_{t+1}\right)$ in D13:        Sample minibatch transitions $\left(s_{t},a_{t},r_{t},s_{t+1}\right)$ from D14:        **if** $s_{t+1}\neq s_{e}$ **then**15:           $y_{i}=r_{i}+\eta \cdot Q\left(s_{i+1},\arg\max_{a}Q\left(s_{i+1},a,\theta \right);\theta^{-}\right)$16:        **else**17:           $y_{i}=r_{i}$18:        **end if**19:    Perform a gradient descent step on the loss value computed according to Equation (13) and update the parameters θ20:        Reset $\hat{Q}\leftarrow Q$ every C steps21:        $s_{t}\leftarrow s_{t+1}$; // go to next step22:        $R_{k}=R_{k}+r_{t}$; // update local greward23:        **if** the task cannot be completed **then**24:           Tasks are not assigned to workers, update status of tasks and workers’set25:        **else**26:           Assign the task to worker, update status of tasks and workers’ set27:        **end if**28:        **if** $R_{k}>R$ **then**29:           $R=R_{k}$; // update global maximum greward30:           Record p,d,t31:           Record paths $\left\{F_{j}:\forall w_{j}\in W\right\}$32:        **end if**33:    **end while**34:
**end for**
35:**return** R, {p,d,t} and $\left\{F_{j}:\forall w_{j}\in W\right\}$


Algorithm 1 shows the implementation details of the D3QN solution. The initialisation of the Q-network, target network and experience pool is performed in lines 1–3, respectively. Line 4 establishes a variable to track the global maximum reward. Platform profit, worker travel distance and worker response time are initialised in line 5. The variable ‘k’ in line 6 keeps track of the current episode count, and learning continues until a termination condition is met. In each episode, the local variables ‘greward’ and the start state are initialised (lines 7–8). If the current state is not a terminal state within the episode (line 9), an ε–greedy strategy is employed to select an action (line 10). The ε–greedy policy chooses the action with the highest Q-value greedily with probability ε; otherwise, a random action is chosen. Upon executing the selected action, the platform observes the reward and the next state (line 11). To reduce data sample correlation, the agents’ experience $\left(s_{t},a_{t},r_{t},s_{t+1}\right)$ is stored in the experience pool (line 12). At each learning step, the platform randomly selects small batches of samples from the experience pool (line 13) and updates the Q-network parameters θ by minimising the mean-squared loss function as defined in Equation (13) (lines 14–19). After a certain number of learning steps, the target network is periodically updated with the parameters from the Q-network (line 20). Line 21 denotes the update of the current state, while line 22 credits the reward to the local agent. The server then evaluates whether the task can be completed and updates the environment (lines 23–27). If the local reward exceeds the global reward (line 29), the global reward is updated (line 30). Profit, driving distance and response time are recorded in line 31, and the worker’s trajectory is stored in line 32. Finally, the function returns the final result in line 35.

### 4.4. Baseline Solutions

For the performance comparison of D3QN, we consider three baseline solutions for the task allocation problem: DQN, ε–greedy, and random solutions. DQN is a well-known algorithm in deep reinforcement learning that has been successfully applied to various combinatorial optimisation problems. It leverages neural networks and Q-learning to interact with the environment and make decisions in order to discover the optimal strategy.

Additionally, greedy-based solutions are commonly employed in previous studies. Hence, the ε–greedy solution serves as a baseline approach. In this solution, the platform selects the worker with the highest greward with probability ε, while random selection is used otherwise. Algorithm 2 illustrates the ε-greedy solution. Furthermore, the random solution corresponds to the scenario where ε is set to 0, implying completely random worker selection.

**Algorithm 2:** ε–Greedy Solution
**Input:** 
V (tasks), W (users), ε (probability of random selection), M (maximum number of episodes with no improvement)**Output:** 
R (greward of all episodes), {p,d,t} (profit of platform, travel distance of workers and workers’ response time), {$F_{j}:\forall w_{j}\in W$} (travelling trajectory of workers)
1:R←−∞; // global maximum greward2:p,d,t←0; // profit, distance and response time3:k←0; // number of episodes with no improvement4:**while** k<M **do**5:    $R_{k}\leftarrow 0$; // local greward in current episode6:    **for** $\forall v_{i}\in V$ **do**7:        **if** there exist feasible users for task $v_{i}$ **then**8:           Random selection with probability ε; otherwise, greedy selection (reward)9:           Get reward of this assignment as $R_{i}$10:           $R_{k}=R_{k}+R_{i}$; // update local greward11:           **if** the task cannot be completed **then**12:               Tasks are not assigned to workers, update status of tasks and workers’ set13:           **else**14:               Assign the task to worker, update status of tasks and workers’ set15:           **end if**16:        **end if**17:    **end for**18:    **if** $R_{k}>R$ **then**19:        R = $R_{k}$; // update global maximum greward20:        Record p,d,t21:        Record paths $\left\{F_{j}:\forall w_{j}\in W\right\}$22:        k = 0; // reset k with improvement23:    **else**24:        k = k + 1; // increase k without improvement25:    **end if**26:
**end while**
27:**return** R, {p,d,t} and $\left\{F_{j}:\forall w_{j}\in W\right\}$


## 5. Numerical Results and Discussion

In this section, the experimental scenario and the parameters of the neural network are set. Additionally, we experimentally evaluate the performance of the D3QN-based solution and the baseline solution. More specifically, the performance of the D3QN-based solution is demonstrated in three scenarios with different numbers of workers.

### 5.1. Simulation Settings

**Figure 3:**
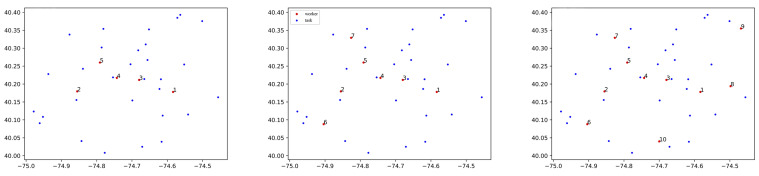
Simulations of scenarios with different number of users.

In our simulations, we used a real dataset from an existing application: Foursquare [60]. Specifically, two files from this data subset were used: (1) the venues’ file representing the location of the tasks and (2) a users’ file corresponding to the location of the workers. The dataset contains 1,021,966 check-in records for 2,153,469 users at 1,143,090 venues over a given time period. To facilitate data processing, the coordinate ranges of the dataset were narrowed down to [−74.9831° W, −74.4322° W] and [40.0023° N, 40.4128° N]. In the simulation setup, the sensing area was set as a rectangular area. Then, 30 sensing tasks and some workers were evenly distributed in the area. There were three different scenarios with 5, 7 and 10 workers, as shown in Figure 3. Euclidean distances were used. Each worker’s travel speed was randomly generated from 10 to 50 km/h, with a reward value set to 1 per unit of travel distance and a maximum travel distance set to 30 km. For each sensing task, the time window started from 0 to 30 and ended at 60 (in minutes). The average user budget for a sensing task submission was 50. Finally, all simulation parameters were set as shown in Table 2.

### 5.2. Parameter Settings of DQN and D3QN Neural Networks

It is particularly important to construct DQN or D3QN networks in order to obtain the best results. The key to optimising the performance of the network is to tune the hyperparameters, such as ε, the exploration rate, the number of neurons in each layer of the Q-network and the learning rate. It is worth mentioning that we performed a moving average with a window size of 20 on the 1D data. This technique helps to reduce noise and smooth out fluctuations in the data, providing a clearer representation of the underlying trends or patterns. By using a sliding average window of size 20, the data are effectively averaged over a specific interval, resulting in a more reliable analysis and interpretation of the experimental results.

**Figure 4:**
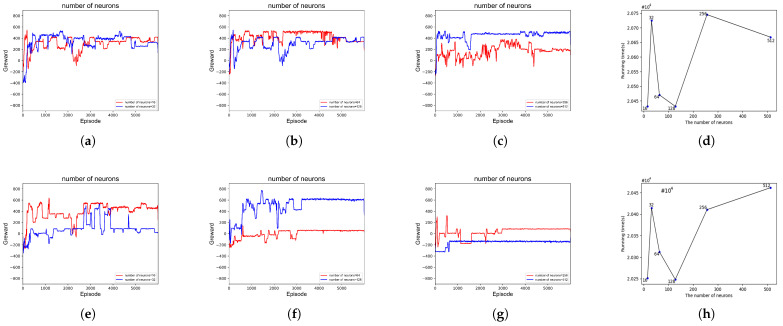
The impact of the number of different neurons on the benefit of platform. (**a**) DQN: The number of neurons is 16 and 32; (**b**) DQN: The number of neurons is 64 and 128; (**c**) DQN: The number of neurons is 256 and 512; (**d**) DQN: Platform operation time; (**e**) D3QN: The number of neurons is 16 and 32; (**f**) D3QN: The number of neurons is 64 and 128; (**g**) D3QN: The number of neurons is 256 and 512; (**h**) D3QN: Platform operation time.

As shown in Figure 4, the number of neurons was chosen in the range of [16–512]. As shown in Figure 4a, when the number of neurons of the DQN was 16 and 32, the network did not have good learning ability due to the relatively small number of neurons. When greater than or equal to 128, as shown in Figure 4c, the complexity of the network structure increased greatly, resulting in slower learning of the network and much higher running time of the platform. This was similar for D3QN, as seen in Figure 4e–g. When the number of neurons is 128, the platform has the shortest running time, the fastest network convergence and can maintain better stability after convergence. Thus, the number of neurons for both networks was chosen to be 128.

**Figure 5:**
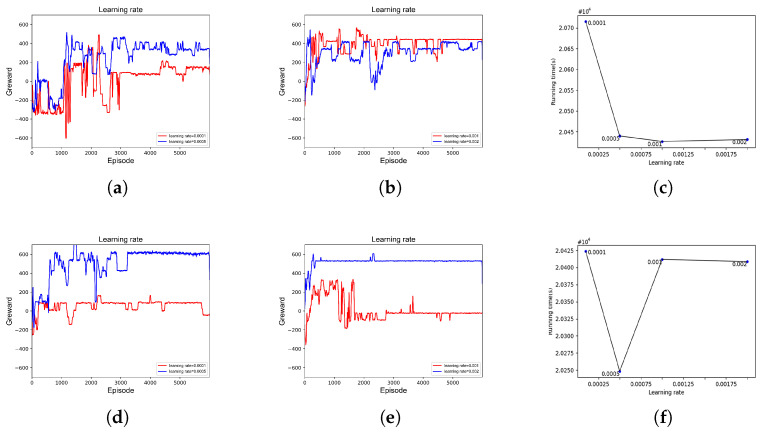
The impact of the different learning rate on the greward of frame. (**a**) DQN: Learning rate is 0.0001, 0.0005; (**b**) DQN: Learning rate is 0.001, 0.002; (**c**) DQN: Platform operation time; (**d**) D3QN: Learning rate is 0.0001, 0.0005; (**e**) D3QN: Learning rate is 0.001, 0.002; (**f**) D3QN: Platform operation time.

As shown in Figure 5, the learning rate increases from 0.0001 to 0.002. As shown in Figure 5a, when the learning rate is 0.0001 or 0.0005, the DQN network structure is relatively simple and the learning rate is low. The network, in this case, does not have good learning ability. Once the learning rate increases to 0.002, the learning effect and convergence speed are accelerated. Moreover, when the learning rate is greater than or equal to 0.0005, the running time of the platforms is shorter and the difference is not significant. For D3QN, as shown in Figure 5d, the convergence effect is best and the platform running time is shortest when the learning rate is 0.0005. Therefore, the learning rates for DQN and D3QN were chosen to be 0.002 and 0.0005, respectively.

Due to space limitations, other parameters are not described. In short, when the replay memory capacity is 50,000, the initial exploration rates are 0.75 and 0.90, the final exploration rate is 0.999, the learning rates are 0.002 and 0.0005, the number of Q-network layers is 2 and the number of neurons in the hidden layer of the Q network is 128, the DQN and D3QN networks tend to be stable and have the best effect. The target network is replaced by the Q-network every 200 learning steps. The discount factor is set to 0.9. Both the Q-network and the target network are DNNs with one hidden layer. Here, Rectified Linear Unit (ReLU) is used in DNN. Finally, the setting of neural network parameters is shown in Table 2.

### 5.3. Ablation Study

To further understand how networks compete with each other in D3QN, we conduct a deeper ablation study of the D3QN algorithm, investigating the true role played by Double DQN, Dueling DQN, and D3QN network structures. In this process, the hyperparameters of the neural network are fixed, the learning rate is 0.0005, the number of neurons is 128, the replay memory capacity is 50,000, the initial exploration rate is 0.90, the final exploration rate is 0.999 and the number of Q-network layers is 2. The number of tasks to be completed is 30 and the number of workers is 5.

The experimental results are shown in Table 3. Compared with the baseline network, the neural network with Dueling DQN increased the Reward value by 27.54%, reaching 600.41; the task completion rate increased by 3.34%, reaching 66.67% and the platform profit increased by 8.99%, reaching 900.88. Response time decreased by 19.42% to 2.99. Compared with the baseline network, the neural network with Double DQN increased the Reward value by 29.36%, reaching 608.99; the task completion rate increased to 66.67%. Platform profits increased by 5.26 to 869.64. The response time decreased by 45.85% to 2.01. Finally, compared with the baseline network, the D3QN neural network using Double DQN and Dueling DQN improved in four indicators, among which, Reward, task completion rate, profit and response time reached 825.24, 76.67%, 986.95 and 1.94, respectively. Experimental results show that Double DQN and Dueling DQN improve the learning efficiency of the neural network, suppress the interference of noise and improve the performance of the neural network.

### 5.4. Result of Greward

**Figure 6:**
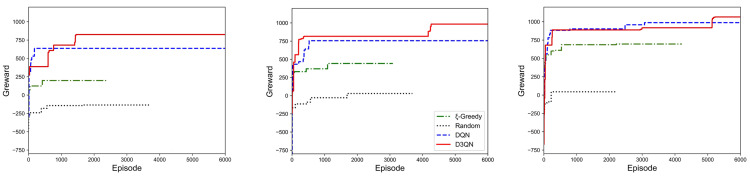
Greward in simulated scenarios with different numbers of users.

Greward is the target of the task allocation problem in (7a)–(7f) and is, therefore, the most important indicator of effectiveness. Figure 6 shows the maximum greward value under all episodes, i.e., the cumulative maximum reward value found before the current episode. From Figure 6, we can observe that the greward of the random solution is the smallest of all the solutions, due to the fact that it does not take into account the suitability of the workers. The traditional greedy-based algorithm is also obviously inferior to deep reinforcement learning methods in terms of learning speed and effectiveness. Ultimately, the D3QN solution achieves the largest greward in all three scenarios. The D3QN solution rapidly increases the greward value of the platform at the initial time and then gradually converges to the final result, demonstrating its excellent learning capability. The curves in Figure 6 clearly show when the D3QN solution outperforms the baseline solution, and the growth curve of the D3QN solution demonstrates its ability to solve the task allocation problem.

### 5.5. Result of Completed Tasks

**Figure 7:**
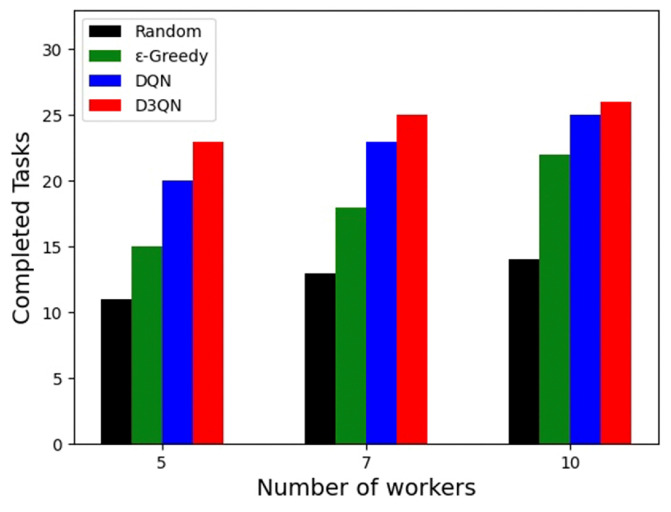
Numbers of completed tasks.

The completion rate of tasks is an important evaluation metric for platform performance and directly affects the platform’s profit and ability to attract users. Here, some tasks were left incomplete. This failure was due to either platform response timeout or the platform algorithm’s inability to select suitable workers who could complete the tasks within the tasks’ response time. Additionally, objective factors such as worker speed or distance between workers and tasks could prevent task completion within the tasks’ response time. In such cases, the server does not actually assign the tasks to the workers but instead marks them as idle, and the tasks are marked as failed. The system then proceeds to the next round of task assignment. This means that task execution failures are not taken into account, and, once a worker accepts a task, they are considered available for task completion. Figure 7 shows the number of tasks completed in the three scenarios. It can be seen that, although the number of tasks completed by all algorithms increases with the number of workers, in the three simulation scenarios with the same number of workers, the random algorithm has the least number of tasks due to its randomness, and the task completion rate of the ε–greedy algorithm is much lower than that of the DQN and D3QN schemes of deep reinforcement learning due to insufficient algorithm capabilities. Among them, the performance of the D3QN algorithm is higher than that of DQN, and the number of completed tasks is also more than that of DQN. Among all algorithms, the number of tasks completed by the D3QN algorithm is the largest. In the D3QN solution, 5, 7 and 10 workers completed 23, 25 and 26 sensing tasks, respectively. Although not all tasks can be completed, many constraints are taken into account, such as randomness of task locations, constraints on worker travel distance and speed, time constraints in completing tasks, etc. Under the constraints of various conditions, the solution of the algorithm may already be the optimal solution of the problem. Finally, it can be seen that the D3QN-based solution accomplishes the most tasks in all simulated scenarios. Therefore, the D3QN-based solution outperforms other solutions in terms of task completion rate.

### 5.6. Result of Platform Profit

**Figure 8:**
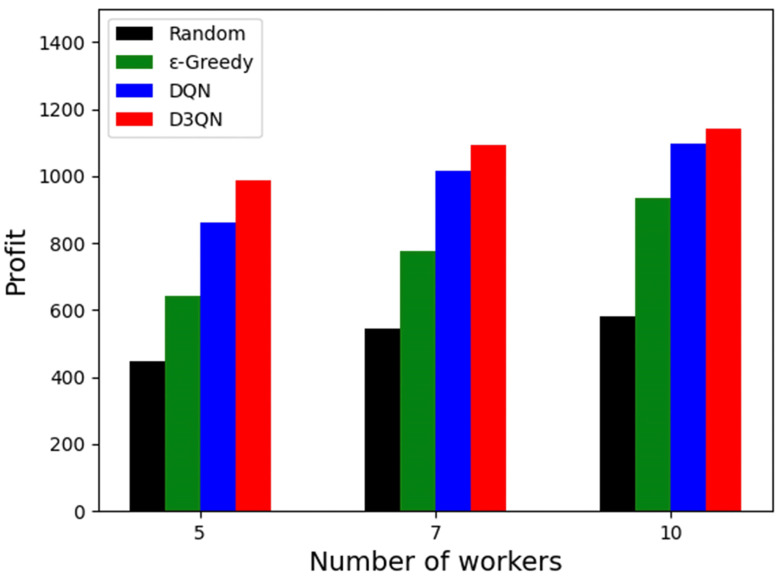
Platform profit.

Platform profit is often an important objective in the task allocation problem, and Figure 8 shows the platform profit for three scenarios with different algorithms. As can be seen, the platform profit increases as the number of workers increases. This is due to the fact that more workers are involved, more paths can be planned and workers’ previous travel paths are optimised, allowing more tasks to be completed while the platform gains more profit. Finally, our proposed D3QN solution achieves maximum profit in all three cases with 986.9, 1091.46 and 1141.23. However, the number of workers has little effect on the random solution with profits of 449.22, 545.31 and 582.82 for the three cases. We also observe that the gap between the DQN and D3QN solutions becomes smaller as the number of workers increases, with 127.32 (5 workers), 79.25 (7 workers) and 44.74 (10 workers). The reason for this is that redundant workers make it less difficult to find a satisfactory solution and the platform can easily find the right workers for the sensing task at a lower cost. Therefore, the DQN solution with the slightly weaker learning ability is close to the D3QN solution in terms of profit as the number of workers increases.

### 5.7. Result of Average Response Time of Workers

**Figure 9:**
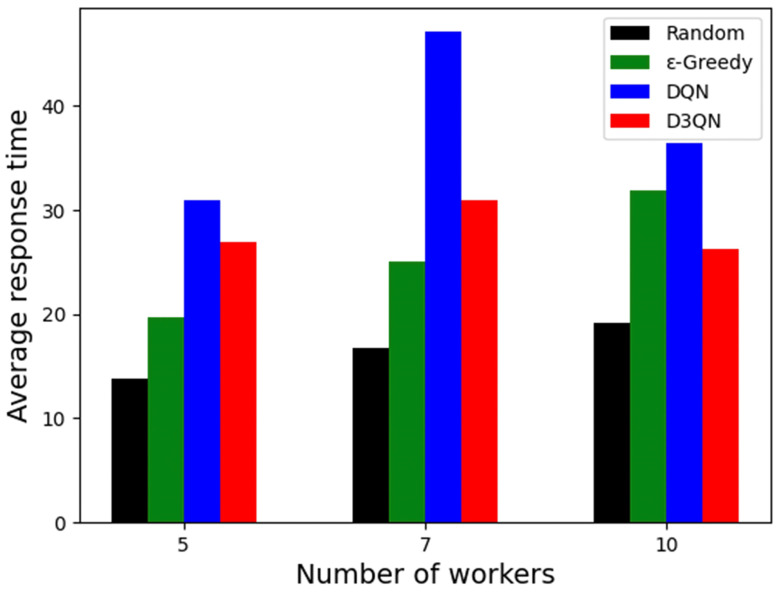
Average response time of workers.

The workers’ response time is an important part of the performance of the task allocation framework. On the one hand, workers’ response time can be saved, allowing for more tasks to be completed and greater profits to be made. On the other hand, when data requesters experience shorter response time for their tasks, it helps to improve their experience and, thus, attract more users to the platform. Here, due to the different number of tasks completed by each method, the time spent is also different. To illustrate, Figure 9 shows the average response time in minutes for workers in the three scenarios. Using the average response time to judge the effectiveness of the methods, we observe that the random algorithm has the smallest response value in all three scenarios, due to the fact that it completes the least number of tasks and does not need to pick the right worker in the random case. Furthermore, the average response time of the DQN solution is the longest among the solutions. In addition, compared to other algorithms, the D3QN solution achieves a shorter response time value in all scenarios.

### 5.8. Result of Average Traveling Distances of Workers

**Figure 10:**
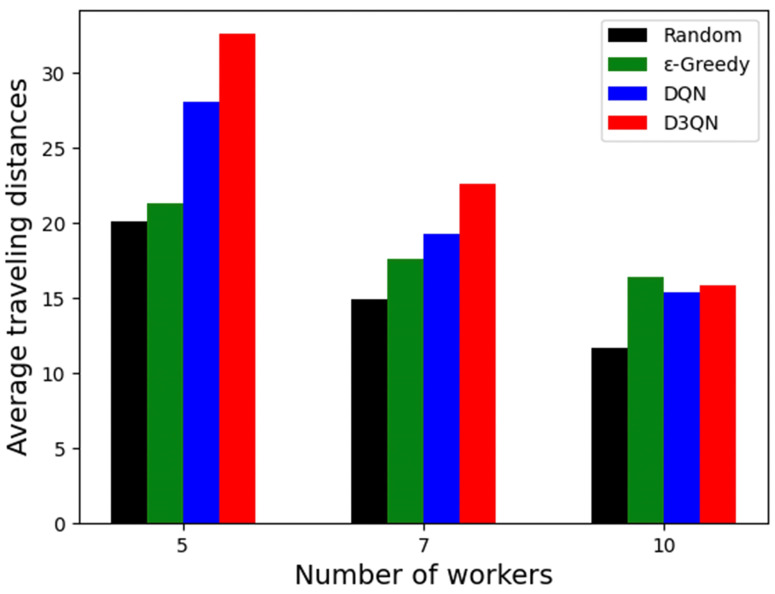
Average traveling distances of workers.

Regarding travel distance, the platform and the workers have the same objective and want shorter travel distances. Figure 10 shows the average travel distance of workers in the three scenarios. Overall, the average travel distance decreases with the number of workers. However, it is clear that the decrease is greater for D3QN, with the average travel distance of the D3QN solutions for the three scenarios being 32.61, 22.64 and 15.87. This is due to the fact that, in scenarios with a smaller number of workers, a single worker needs to perform a larger number of tasks, which can easily lead to a situation where the optimal solution worker for a task is performing other tasks. This situation is gradually alleviated as the number of workers increases, and, when more workers are involved, fewer sensing tasks are assigned to each worker on average, and then workers can use shorter paths to perform the assigned tasks. As can be seen from the figure, D3QN has the largest decline, and, when the number of workers increases to 10, the D3QN scheme works best. It is worth noting that the D3QN solution achieves the highest profit and task completion numbers as well as a short response time in all scenarios, which shows that D3QN is significantly better than the other comparison algorithms.

## 6. Conclusions

In this paper, a dynamic multi-objective task allocation framework for MCS based on deep reinforcement learning is presented. We build a dynamic multi-objective task allocation model using a Markov decision process. A weighted multi-objective approach is proposed in order to take into account the participant experience while considering the benefits of the platform. To solve the dynamic task assignment problem, a D3QN-based solution is proposed from a decision-making perspective, and its performance is compared with three other baselines (DQN, ε–greedy and random solution). Finally, experimental results on a real dataset show that our proposed solution outperforms the baseline solutions in terms of platform profit, response time and travel distance. As future work, we propose to investigate allocation strategy optimisation when assigning multiple tasks to a single worker. We also propose to investigate higher dimensional task allocation problems and try new deep reinforcement learning methods to optimise the framework performance. 

## Figures and Tables

**Table 1:** Model  parameters.

Task Parameters	
V	Set of tasks
$v_{i}$	Task i
$b_{i}$	Budget of task $v_{i}$
$l_{i}$	Location of task $v_{i}$
$t_{i}$	Time of submission of task $v_{i}$
$t_{r}^{i}$	Response time for task $v_{i}$
$t_{e}^{i}$	End time of task $v_{i}$
Worker parameters	
W	Set of workers
$W_{i}$	Set of selected feasible workers to perform task $v_{i}$
$w_{j}$	Worker j
$f_{j}$	Travelling speed of worker $w_{j}$
$F_{j}$	Travelling trajectory of worker $w_{j}$
$F_{j}^{i}$	Travelling path from worker $w_{j}$ to task $v_{i}$
$d(F_{j}^{i})$	Travelling distance from worker $w_{j}$ to task $v_{i}$
$p_{j}$	Reward of $w_{j}$
$\theta_{j}$	Coefficient affecting the reward of worker $w_{j}$
$l_{j}$	Location of worker $w_{j}$
$t_{j}$	Time that the worker $w_{j}$ starts to preform task
$t_{j}^{i}$	Time that the worker $w_{j}$ starts to preform task $v_{i}$
Other	
$d_{max}$	Maximum travelling distance of workers
B	Total budget
D	Total travelling distance
T	Total response time
P	Total profit
R	Objective function

**Table 2:** Experimental settings.

Parameters	Value
Number of works (w)	[5, 7, 10]
Number of tasks (v)	30
Budget of task	50
Starts of tasks’ time windows	[0, 30]
Ends of tasks’ time windows	60
Intervals of tasks’ time windows	[10, 20]
Number of episodes	6000
Payment per unit distance of workers	1
Workers’ velocity	[10, 50]
Start Probability of random selection in DQN	0.75
Start Probability of random selection in D3QN	0.90
Probability of random selection in ε-Greedy	0.95
End Probability of random selection	0.999
Capacity of the replay memory	50,000
Steps to update the target network	200
Discount factor in DQN	0.9
Discount factor in D3QN	0.9
Learning rate in DQN	0.002
Learning rate in D3QN	0.0005
Size of the hidden layer in DQN	128
Size of the hidden layer in D3QN	128
Size of the minibatch of samples	128

**Table 3:** Ablation experiment of D3QN algorithm applied to crowd sensing task assignment.

Double DQN	Dueling DQN	Reward	Task Completion Rate	Profit	Response Time
×	×	470.80	63.33%	826.43	3.71
×	✓	600.41	66.67%	900.88	2.99
✓	×	608.99	66.67%	869.94	2.01
✓	✓	825.24	76.67%	986.95	1.94

## Data Availability

The data “umn/sarwat foursquare dataset” used in this study are openly available at https://archive.org/details/201309_foursquare_dataset_umn, accessed on September 2021.
